# Muscle wasting in cancer: opportunities and challenges for exercise in clinical cancer trials

**DOI:** 10.1002/rco2.56

**Published:** 2021-12-22

**Authors:** Ciaran M. Fairman, Simon Lønbro, Thomas D. Cardaci, Brandon N. VanderVeen, Tormod S. Nilsen, Angela E. Murphy

**Affiliations:** 1 Department of Exercise Science, University of South Carolina, Columbia, South Carolina 29033, USA; 2 Department of Public Health, Section for Sports Science, Aarhus University, Aarhus, Denmark; 3 Department of Pathology, Microbiology and Immunology, School of Medicine, University of South Carolina, Columbia, South Carolina, USA; 4 Department of Physical Performance, Norwegian School of Sports Sciences, Oslo, Norway

**Keywords:** Muscle loss, Cachexia, Resistance exercise, Sarcopenia

## Abstract

**Background:**

Low muscle in cancer is associated with an increase in treatment-related toxicities and is a predictor of cancer-related and all-cause mortality. The mechanisms of cancer-related muscle loss are multifactorial, including anorexia, hypogonadism, anaemia, inflammation, malnutrition, and aberrations in skeletal muscle protein turnover and metabolism.

**Methods:**

In this narrative review, we summarise relevant literature to (i) review the factors influencing skeletal muscle mass regulation, (ii) provide an overview of how cancer/treatments negatively impact these, (iii) review factors beyond muscle signalling that can impact the ability to participate in and respond to an exercise intervention to counteract muscle loss in cancer, and (iv) provide perspectives on critical areas of future research.

**Results:**

Despite the well-known benefits of exercise, there remains a paucity of clinical evidence supporting the impact of exercise in cancer-related muscle loss. There are numerous challenges to reversing muscle loss with exercise in clinical cancer settings, ranging from the impact of cancer/treatments on the molecular regulation of muscle mass, to clinical challenges in responsiveness to an exercise intervention. For example, tumour-related/treatment-related factors (e.g. nausea, pain, anaemia, and neutropenia), presence of comorbidities (e.g. diabetes, arthritis, and chronic obstructive pulmonary disease), injuries, disease progression and bone metastases, concomitant medications (e.g., metformin), can negatively affect an individual’s ability to exercise safely and limit subsequent adaptation.

**Conclusions:**

This review identifies numerous gaps and oppportunities in the area of low muscle and muscle loss in cancer. Collaborative efforts between preclinical and clinical researchers are imperative to both understanding the mechanisms of atrophy, and develop appropriate therapeutic interventions.

## Introduction

Individuals with cancer can experience muscle loss at an exponential rate, with declines in muscle mass that would typically take decades, occurring in months.^1^ The severity and phenotypic presentation of muscle loss can vary depending on tumour-related and treatment-related factors and subsequent alterations in muscle protein turnover, systemic inflammation, mitochondrial dysfunction, and oxidative stress.^2,3^ However, the accelerated muscle loss has dramatic health consequences, increasing the risk of cancer-related and all-cause mortality while also contributing to reductions in quality of life and physical function.^4–6^ Consequently, there is a clear need for the investigation of strategies to counter cancer-related muscle loss.

Despite an ever-increasing interest in exercise as a therapeutic intervention to counteract cancer-related muscle loss, results from the literature remains decidedly mixed.^7–9^ The reasons for divergent reports on exercise to reverse muscle loss are multifactorial. Ever-evolving definitions and diagnostic criteria of muscle loss (e.g. sarcopenia/cachexia) conditions make it difficult to discern between conditions, their aetiology, prevalence, and ultimately, appropriate interventions.^10,11^ Different results may also be due to differences in tumour type (i.e. patient population); treatment type/burden; and the type, timing, and quality of exercise interventions.^12–14^ In individuals with advanced cancer or aggressive muscle wasting, challenges to recruitment and feasibility of delivering an exercise intervention further limit our understanding of the role of exercise in these settings. Taken collectively, these factors beg the question: Are these potential targets for, or insurmountable barriers to exercise-induced muscle growth? Thus, the purpose of this paper is (i) to review the factors influencing skeletal muscle mass regulation, (ii) provide an overview of how cancer/treatments negatively impact these, (iii) review factors beyond muscle signalling that can impact the ability to participate in and respond to an exercise intervention to counteract muscle loss in cancer, and (iv) provide perspectives on critical areas of future research.

## Factors influencing skeletal muscle mass regulation

### Skeletal muscle mass regulation

Skeletal muscle mass maintenance and growth is a dynamic physiological process incorporating several mechanisms responsible for protein turnover, metabolic homeostasis, and regeneration. The factors involved in the regulation of muscle mass have been well-defined and thoroughly reviewed.^15–17^ However, it is important to understand some of these key regulators to appreciate how cancer/treatments negatively impact them, the resultant impact on both muscle loss and the ability to respond to therapeutic interventions. We will provide a brief overview of the regulatory elements that have been examined with both muscle growth and muscle loss.

### Muscle growth

The role of mammalian target of rapamycin (mTOR) in regulating skeletal muscle mass is well-defined.^17^ One of the upstream regulators of the mTOR pathway is the phosphatidylinositol-3-kinase/protein kinase B (Akt) pathway that is activated by growth factors [i.e. insulin like growth factor 1 (IGF-1)].^18^ There exists two distinct mTOR complexes, mTORC1 and mTORC2, each with diverse signalling mechanisms and functionality.^17,18^ Cell growth, metabolism, and protein synthesis are primarily regulated by mTORC1, whereas mTORC2 instead controls proliferation and survival during cellular stress.^19^ Up-regulated Akt/mTOR signalling further enhances the cell growth and proliferation through the inhibition of GSK-3B,^20^ which is involved in muscle catabolism as discussed below.

Mitochondrial signalling regulates muscle mass and function through its role in ATP production, calcium handling, oxidative-antioxidant balance, and apoptosis—all of which contribute to protein balance in skeletal muscle.^21–25^ Moreover, the process of protein synthesis is extremely energetically costly and requires significant amounts of ATP for charging aminoacyl-tRNAs^26^ along with regenerating GTP for the translation elongation factors responsible for peptide bond formation and ribosome locomotion along mRNA.^27^ The peroxisome proliferator-activated receptor-γ coactivator-1α (PGC-1α) has often been considered a key regulator of mitochondrial homeostasis responsible for expanding the pre-existing mitochondrial network via mitochondrial biogenesis.^28^ This involves synthesizing, importing, and incorporating proteins to the existing mitochondria in order to increase the capacity of these cellular functions. Expansion of this mitochondrial network contributes to altering skeletal muscle health and function through enhancing oxidative capacity, increasing fatty acid β-oxidation, attenuating muscle glycogenolysis, delaying the onset of muscle fatigue, and improving muscle performance.^29,30^ The energy sensing enzyme, 5’ AMP-activated protein kinase (AMPK), is the most notable PGC-1α activator; however, other growth factors such as androgens and IGF-1 were also reported to induce PGC-1α signalling.^31,32^

Satellite cells are involved in repair and growth of skeletal muscle through the provision of new myonuclei that provide the necessary machinery for muscle remodelling and eventual hypertrophy. Satellite cells primary role in muscle mass regulation is the (re)generation of new/repaired fibres.^33^ It has been hypothesized that repeated mechanical load through resistance exercise causes damage to muscle sufficient to activate satellite cells. Activated satellite cells can then proliferate and fuse with myofibres to support remodelling and hypertrophic processes.^34,35^ Emerging evidence suggests skeletal muscle repair and remodelling following exercise-induced muscle damage also activates a self-repair mechanism independent of satellite cells. Specifically, a signalling cascade involving calcium, cell division control protein 42 homologue, and phosphokinase C triggers myonuclear migration to the damaged area within the muscle and encourage sarcomere repair and delivery of mRNA for muscle cell reconstruction.^36^

Mechanical signals through muscle contraction have also been implicated in skeletal muscle growth, although details of specific mechanisms remain underdeveloped. Mechano-sensors allow skeletal muscle fibres to detect mechanical signalling with resistance exercise to initiate anabolic signalling.^15^ Integrins, titin, and filamin-C-Bag3 are all suggested to respond to different mechanical stimuli (contraction force, cytoskeleton loading, and mechanical properties of the extracellular matrix) to promote muscle growth.^37,38^ While molecular pathways are still being uncovered, inactivity and periods of unloading may contribute to muscle loss, during disuse or disease, at least partly due to reduced signalling through these pathways.

### Catabolism

The ubiquitin proteasome system (UPS) is the primary signalling pathway responsible for myofibrillar and sarcomeric skeletal muscle protein degradation.^39^ Ubiquitination of the target protein is regulated by three enzymes: ubiquitin-activating enzyme (E1), ubiquitin-conjugating enzyme (E2), and ubiquitin-ligase enzyme (E3). E3 ubiquitin-ligase enzymes are the key enzymes in this process due to their ability to control substrate-specific proteolysis through recognition of the specific protein substrate and transfer of the activated ubiquitin.^40,41^ Muscle RING finger-containing protein 1 (MuRF1) and muscle atrophy Fbox protein (MAFbx/Atrogin1) are the most commonly studied E3 enzymes for their known ability to degrade a number of regulatory and functional proteins in skeletal muscle.^42^ Their activity is regulated largely by the translocation and activity of FoxO transcription factors, which are required to increase MuRF1 and MAFbx expression.^40,41^

The autophagy-lysosome system is the principal mechanism for degrading long-lived organelles and large protein aggregates or damaged proteins. Physiologically, autophagy is critical for muscle health and homeostasis through regulating cellular quality control and proper protein/organelle turnover. Regulation of the autophagy-lysosome degradation pathway is influenced by a variety of metabolic factors, transcriptional and microRNA-based regulators, and chaperone proteins. mTOR and IGF-1/insulin signalling, calpain activity, and nutrient levels, along with transcriptional regulators can all influence autophagy, and lysosomal genes and protein expression critical to the function of this system.^43,44^

Myostatin is a member of the transforming growth factor-β superfamily and has been shown to be a potent negative regulator of muscle mass.^45,46^ Myostatin’s negative influence on skeletal muscle mass is suggested to be due to its negative impact on AKT signalling, myoblast differentiation, and down-regulation of genes involved in muscle homeostasis.^45^ Additionally, myostatin activates AMPK during energy stress to increase nutrient availability.^47^ Collectively, myostatin’s ability to interact with these other important regulatory and signalling proteins demonstrates its significant roles in the multiple processes in skeletal muscle including regulation of protein homeostasis, glycolysis, and mitochondrial function.^47,48^

Nuclear factor-κB (NF-κB) is one of the primary transcriptional mediators responsible for elevating muscle proteolysis through the up-regulation of key E3 ligases and inhibition of protein synthesis.^49,50^ Elevations in pro-inflammatory cytokines such as tumour necrosis factor α (TNF-α), interleukin (IL)-1β, and IL-6 into circulation and interactions with their respective receptor induce a cascade of signalling events leading to elevations in NF-κB activity and eventual increases in proteolytic gene and protein expression. Increased NF-κB activation and translocation into the nucleus increases atrophy-related gene expression, namely, MAFbx and MuRF1.^51,52^ Moreover, TRADD, an intermediary adaptor protein, interacts with the TNFR1 and recruits other key regulatory proteins such as TRAF2, RIP, and FADD all of which play important roles in mediating NF-κB signalling and facilitating muscle catabolism.^51,52^

Collectively, the regulation of skeletal muscle mass is coordinated by a variety of factors including hormones, nutrient availability, mechanical stimuli, and inflammation. Disruptions to these pathways in ageing, disuse, and/or disease result in an imbalance between the anabolic and catabolic processes of skeletal muscle, accelerating atrophy. Understanding the regulation of skeletal muscle mass can improve the consideration of how cancer/treatments contribute to muscle loss and potentially identify targets for therapeutic interventions.

## How cancer and its treatments impact muscle loss

The specific mechanism(s) of muscle loss is likely to vary both by tumour-derived signalling molecules and treatment elements (e.g. treatment type, treatment dose, and multimodal combinations) in addition to individual characteristics and circumstances. Here, we provide an overview of the influence of tumour and different treatments on skeletal muscle loss.

### Cancer-related muscle catabolism

The understanding of the catabolic effects of cancer, independent of its treatments, is ever growing. The UPS and disrupted autophagy have been identified as primary candidates of catabolism in cancer, mediated by pro-inflammatory factors such as IL-6, TNF-α, and IL-1. Further, it was recently demonstrated that myogenic signalling (lower mRNA contents of MyoD, CyclinD1, Myogenin, and Pax7) was suppressed in tumour-bearing mice relative to control animals.^53^ Fractional synthetic rates were decreased by 40% in tumour-bearing mice relative to controls, 4 weeks after implantation.^53^ Lastly, the role of mitochondrial dysfunction in skeletal muscle loss is being increasingly recognized, although exact mechanisms have yet to be fully elucidated. Reductions in muscle protein synthesis through the activation of AMPK cascade and resulting suppression of mTORC1, decreased mitochondrial biogenesis (through decreased activation of PGC-1α, SIRT-1, and NRF-1) and the generation of excess reactive oxygen species (ROS), are suggested as potential contributors to mitochondrial dysfunction and muscle loss.^54^ These aberrations in mitochondrial function are likely to be mediated by high-grade inflammation.^54^ These pre-clinical insights highlight that cancer alone can induce catabolism, specifically through a combination of increased markers of protein breakdown in the UPS, reductions in muscle protein synthesis, and metabolic dysfunction.

### Cancer treatments

The primary candidate and more commonly studied modality for muscle loss in pre-clinical studies is chemotherapy. However, individuals with cancer often receive a variety of treatments that exert differential effects on muscle function, signalling, and morphology. Moreover, more than one chemotherapy agent is often delivered, or chemotherapy in concert with other treatments such as radiation, which could exacerbate these effects. In this section, we provide a brief review of the impact and suggested mechanisms by which these treatments impact muscle loss.

### Chemotherapy

#### Cisplatin

Cisplatin is a cytotoxic chemotherapeutic agent commonly used in a variety of tumour types, including bladder, ovarian, pancreatic, and lung cancers.^55^ The UPS and autophagy-lysosome pathways have been proposed as core components of cisplatin-induced muscle dysfunction, which is mediated by the activation of E3 ligases, MuRF, and MAFbx/atrogin-1 and expression of autophagy-related genes, respectively.^56–58^ Cisplatin administration is also associated with a reduction in the expression of MyoD and myogenin (stimulators of muscle regeneration) and an up-regulation of myostatin that inhibits the mTORC1 pathway, contributing to catabolism. Lastly, cisplatin is associated with an increase in the NF-kB pathway (mediated by the expression of inflammatory cytokines such as TNF-α and IL-1), which further contributes to muscle loss.^58,59^

No study to date has examined the clinical impact of cisplatin on muscle loss. However, Christensen *et al*. examined the safety and efficacy of resistance training in individuals with germ cell cancer receiving cisplatin-based combination chemotherapy [bleomycin–etoposid–cisplatin (BEP)].^60^ Individuals were assigned to resistance exercise or usual care for 9 weeks during BEP. The primary endpoint of muscle fibre size (evaluated as cross-sectional area from muscle biopsies taken from the vastus lateralis) did not change substantially, regardless of group allocation. The secondary outcome of whole-body lean mass (assessed by dual-energy X-ray absorptiometry) decreased by 2.56 kg in the usual care group compared with only a 1.34 kg loss in the resistance training group. Exploratory analysis revealed that muscle mRNA expression of markers of muscle growth (GAPFH, IGF-1Ea, and IGF-1Ec), atrophy (Atrogin1 and MURF1), inflammation (TNF-α, TNFR1, and TNFR2), and protein degradation (UBC, PSMA2, CTSB, and CAMK2B) were relatively unaffected by BEP; however, both IL-6 and myostatin were increased.^61^ This study had a small sample size, in addition to several muscle biopsy samples deemed not valid for analysis of the primary outcome of fibre size. The authors also acknowledged several limitations, including the number of statistical tests and a lack of blinding of outcome assessors. Consequently, this precludes any conclusive understand of the impact of cisplatin on skeletal muscle loss and the role of exercise as a countermeasure.

#### Doxorubicin

Doxorubicin is another common chemotherapy agent used as a treatment for a variety of cancers including breast, ovary, bladder, and certain leukaemias and lymphomas.^62^ The UPS and autophagy-lysosome pathways have both been proposed as contributors to skeletal muscle atrophy with doxorubicin.^63^ Additionally, oxidative stress as a result of mitochondrial dysfunction has also been proposed as a contributor to doxorubicin-induced atrophy.^64^ Specifically, the accumulation of doxorubicin in the mitochondria of skeletal muscle increased the production of ROS (via formation of superoxide radicals from the reduction of doxorubicin by NADH-dehydrogenase), increasing mitochondrial degradation and myofibrillar protein degradation (through the activation of proteases calpain-1 and caspase-3).^65,66^ The administration of doxorubicin is associated with a reduction in mitochondrial respiration, with a decrease in maximal ADP-stimulated respiration supported by complex 1 (pyruvate/glutamate) and complex II (succinate) substrates.^64,67^ Further, doxorubicin increases the rate of mitochondrial H_2_O_2_-emitting potential, supporting the mitochondrial-induced increase in ROS production.^68^ Collectively, these findings indicate how mitochondrial dysfunction through chemotherapy may contribute to the loss of muscle mass in individuals with cancer. Lastly, disruptions in the insulin signalling pathway may decrease the expression of proteins involves in glucose uptake (i.e. GLUT4 and AMPK), reducing protein synthesis and contributing to an overall catabolic state.^69^

#### Fluorouracil

Fluorouracil (5-FU) alone and in combination with other chemotherapeutic agents have been used for the treatment of a variety of cancers such as colorectal, breast, and head and neck cancer.^70^ In fact, combination chemotherapy is increasingly common, where multiple chemotherapeutic agents are given together in the treatment of cancer. This combination approach may result in distinct and differential side effects. For example, Barretto *et al*. recently compared the effects of the administration of FOLFOX (5-FU, leucovorin, and oxaliplatin) to FOLFIRI (5-FU, leucovorin, and irinotecan) for 5 weeks on body weight, muscle mass, and strength in mice.^71^ Animals treated with FOLFIRI experienced extensive weight loss during the last 2 weeks of treatment, whereas animals treated with FOLFOX maintained body weight. Further, the administration of different regimens was associated with differential patterns of muscle loss (all chemotherapy treated animals underwent a loss in quadriceps muscle mass, whereas only animals treated with FOLFIRI experienced a loss in gastrocnemius and tibialis mass). Muscle loss was associated with up-regulation of ERK1/2 and p38 MAPKs but no changes in UPS activity or expression of transforming growth factor-β.^71^ Further, FOLFIRI was associated with a reduction in pAKT (whereas FOLFOX was not). Combination chemotherapy (cyclophosphamide, doxorubicin, and 5-fluorouracil) was also demonstrated to be associated with a loss of muscle mass mediated by increases in MAFbx, MuRF1, and FoxO1 expression and activation of the UPS.^72^ These indicate that both FOLFIRI and FOLFOX contribute to muscle catabolism, although may have differing mechanistic contributions. While 5-FU alone has not been demonstrated to reduce skeletal muscle mass in mice, disruptions to the skeletal muscle microenvironment—loss of immune cells—has been suggested to reduce skeletal muscle plasticity.^73^

### Clinical trials in combination chemotherapies

There is a paucity of clinical trials investigating the impact of various chemotherapy agents on skeletal muscle mass, or exercise as a countermeasure. Møller *et al*. recently examined molecular and cellular adaptations to chemotherapy and exercise training in 10 women with cancer (breast, *n* = 7; head and neck, *n* = 1; rectal, *n* = 1; and sarcoma, *n* = 1).^74^ Phosphorylation of AMPK did not change during the study, while ACC (a substrate of AMPK) increased during chemotherapy and remained stable during exercise with chemotherapy. Additionally, the number of satellite cells remained unchanged. Interestingly, chemotherapy and exercise training did not change protein expression of mitochondrial proteins (Cyt-C, COX-IV, PDH, SDHA, and VDAC). Further, protein expression of MURF1 and Atrogin1 decreased during chemotherapy administration and increased again with exercise during chemotherapy. The authors concluded that various skeletal muscle signalling pathways are dysregulated during chemotherapy administration and that exercise training may be able to prevent further disruption. However, participants in the study were all receiving combination chemotherapies (i.e. doxorubicin with epirubicin, oxaliplatin with 5-fluorouracil, or others), limiting the ability to distinguish between the impact of individuals chemotherapy agents. Importantly, this study was in 10 individuals, with biopsies taken from ten, nine, and six participants at baseline, post-test, and follow-up, respectively. Consequently, the small sample size with heterogeneous cancer types (and treatment schedules) limits our ability to interpret anything conclusive from these data.

Mijwel *et al*. conducted a randomized controlled trial comparing the effects of moderate-intensity aerobic training combined with high-intensity interval training, resistance training combined with high-intensity interval training, or usual care in women with breast cancer undergoing anthracycline, taxane, or combination (anthracycline, taxane, and/or herceptin) chemotherapy.^75^ Muscle fibre cross-sectional area, capillaries per fibre, myosin heavy-chain isoform type 1, and citrate synthase activity declined in individuals receiving usual care, whereas individuals in both exercise groups were able to counteract these declines. The usual care group demonstrated lower levels of PINK1 (a marker of mitophagy), whereas both exercise groups remained stable. There was an increase in satellite cell count per fibre in resistance training combined with high-intensity interval training relative to usual care and aerobic training combined with high-intensity interval training. These results indicate that exercise may preserve or improve markers of skeletal muscle function and morphology relative to usual care in individuals with breast cancer undergoing different chemotherapy combinations. However, this study has some concerns regarding the risk of bias, including the allocation of participants, blinding of outcome assessors, and incomplete outcome data. Further, the small sample size and large number of tests preclude any definitive understanding of the impact of exercise on muscle signalling during the receipt of chemotherapy. As such, caution should be exerted when interpreting these study results.

There is a clinical trial ongoing (NCT03291951) examining the impact of home-based resistance exercise on treatment toxicity in colorectal cancer patients undergoing chemotherapy.^76^ Recruited participants will be receiving one of the following regimens: FOLFOX [capecitabine and oxaliplatin (CAPOX)] or capecitabine.^76^ Although muscle mass is not a primary endpoint in this trial, the results will provide information on the impact of participation in resistance exercise in mitigating treatment-related toxicities in individuals with colorectal cancer undergoing chemotherapy.

### Glucocorticoids

Corticosteroids have a variety of therapeutic uses in cancer treatment settings. High-dose corticosteroids are used in conjunction with chemotherapy to suppress acute infusion-related reactions such as pain, nausea, and vomiting,^77^ and also as a direct component of therapy in a variety of malignant lymphoid cancers (e.g. acute lymphoblastic leukaemia and Hodgkin’s lymphoma) to promote cell apoptosis.^78^ Furthermore, high-dose corticosteroids remain central to the management of symptomatic malignant disease affecting the neurologic system including primary and secondary brain tumours, as well as co-medications in pain management.^78,79^

Glucocorticoids such as prednisone and dexamethasone exert their clinical effect through binding to glucocorticoid receptors to up-regulate the expression of anti-inflammatory proteins, down-regulate pro-inflammatory proteins and affect the production of cell adhesion molecules and other key enzymes involved in the host inflammatory response.^80,81^ Corticosteroid administration may contribute to skeletal muscle atrophy/myopathy in a dose-dependent fashion, with fast-twitch glycolytic fibres particularly susceptible.^82,83^ Dexamethasone stimulates the expression of a protein regulated in development and DNA responses-1 (REDD1), which is a potent inhibitor of mTOR.^84^ Further, glucocorticoids activate the UPS and results in increases in MAFbx and MuRF-1 mRNA with concurrent skeletal muscle atrophy.^85,86^ Glucocorticoid may also impair glucose metabolism, interference with insulin signalling cascade, including beta cell dysfunction,^87^impaired insulin release,^88^ decreases insulin sensitivity,^89,90^ and reduced peripheral uptake at the tissue.^91^ Thus, the catabolic effects of corticosteroids on skeletal muscle are likely due to impaired glucose uptake into cells, the stimulation of muscle protein breakdown, and inhibition of muscle protein synthesis.^83^ To our knowledge, there has been no clinical investigation of the impact of glucocorticoids on skeletal muscle morphology in cancer or the use of exercise as a countermeasure.

### Tyrosine kinase inhibitor therapy

Tyrosine kinase inhibitor (TKI) therapy has been approved for clinical use in a variety of cancers, including renal cell carcinoma, chronic myeloid leukaemia, and certain breast and lung cancers.^92^ Tyrosine kinases are enzymes that target proteins involved in normal cellular processes. Their receptors on the cell surface link extracellular signals to the cytoplasm.^93^ The overexpression of kinases results in uncontrolled blood vessel formation, cell growth, and malignancy. Further, tyrosine kinases receptors are associated with the overactivation of the phosphatidylinositol-3-kinase/AKT/mTORC1 pathway, which can reduce cell apoptosis and promote cell proliferation.^94^ The inhibiting of this signalling has downstream effects on regulatory elements of muscle mass, supported by the growing body of evidence demonstrating a loss of muscle mass in individuals receiving different TKIs.^95,96^ By targeting the tyrosine receptor, TKIs can indirectly suppress the AKT/mTORC1 pathway, reducing its impact on protein synthesis and muscle growth.^94^ It has also been suggested that, in the instance of Sorafenib, the RAS/Raf/MEK/ERK signalling pathway could be inhibited with TKIs, inhibiting muscle growth.^94^

There is a paucity of clinical research investigating the impact of TKIs on muscle cellular morphology and function. Janssen *et al*. recently conducted a cross-sectional study in individuals with chronic myeloid leukaemia (*n* = 20) compared with apparently healthy controls (*n* = 20).^97^ Increases in skeletal muscle fatigability and reductions in maximal force relative to non-cancer controls were observed. Analysis of resting muscle biopsies revealed no differences in mitochondrial function ([1-^14^C]-pyruvate oxidation rates or ATP production rate). Much more research is needed to understand the impact and potential reversibility of skeletal muscle dysfunction with TKIs in cancer.

### Hormonal therapy for prostate cancer

Androgen deprivation therapy (ADT) is a common hormone therapy for controlling the growth and spread of prostate cancer.^98,99^ Despite its clinical success in treating the disease, its administration results in a reduction in the activity/concentration of testosterone to castration levels. The resulting hypogonadal condition is associated with the accumulation of fat mass and reductions in bone mineral density and muscle mass.^100^ A rate of ~3% loss in total lean mass has been reported as early as 26 weeks following treatment.^98–100^ This loss is compounded by the results of a recent meta-analysis, demonstrating that exercise was not able to increase muscle mass in men diagnosed with prostate cancer receiving ADT.^8^ Consequently, the deleterious effects of hormone therapy and the resultant castrate condition may compound the challenges to increasing muscle mass in this population.

Nilsen *et al*. conducted one of the only studies to date investigating muscle directly with resistance training in prostate cancer.^101^ Individuals with prostate cancer receiving ADT participated in 16 weeks of either resistance training or usual care. There was an increase in knee extensor strength and cross-sectional area of type II muscle fibres with resistance training, although the number of satellite cells per fibre did not change, contrary to their hypothesis. However, the increase in cross-sectional area type II fibres was lower than what is typically seen with older adults, in addition to the absence of training effect of satellite cells may partially explain the blunted response to training. Ultimately, much more work is needed to understand the impact of cancer treatments on muscle cellular outcomes in prostate cancer and how this impacts the response to exercise training.

### Radiotherapy

Radiation therapy is used in the treatment of many cancers, including breast, prostate, head and neck, and lung cancer. Radiation-induced fibrosis in skeletal muscle is a common occurrence in cancer, resulting in impairments in regeneration and growth.^102^ Whether this effect is localized specifically to the site of radiation and if it has an impact on response to hypertrophic stimuli in humans is yet to be discovered. Additionally, despite its occurrence, the underlying physiological mechanisms are still unclear. One hypothesis is that satellite cell depletion occurs with radiation, primarily through breaks in strands of the cell’s DNA.^103,104^ An emerging body of pre-clinical evidence indicates that irradiation-induced satellite cell depletion impairs the response to hypertrophic stimuli.^104,105^ Further, it appears as though the impact of radiation on skeletal muscle morphology might be dose dependent and influenced by fibre type. In 2014, Hardee *et al*. demonstrated muscle protein and RNA content were reduced with a single 16 Gy dose of radiation, but not with four fractionated doses of 4 Gy.^106^ Further, their results indicated that type IIB myofibres were susceptible to radiation, whereas type IIa myofibres were not affected. The authors conclude that the therapeutic dose of radiation may impact various myofibre types differently.^106^

### Summary of cancer treatments on skeletal muscle loss

**Figure 1:**
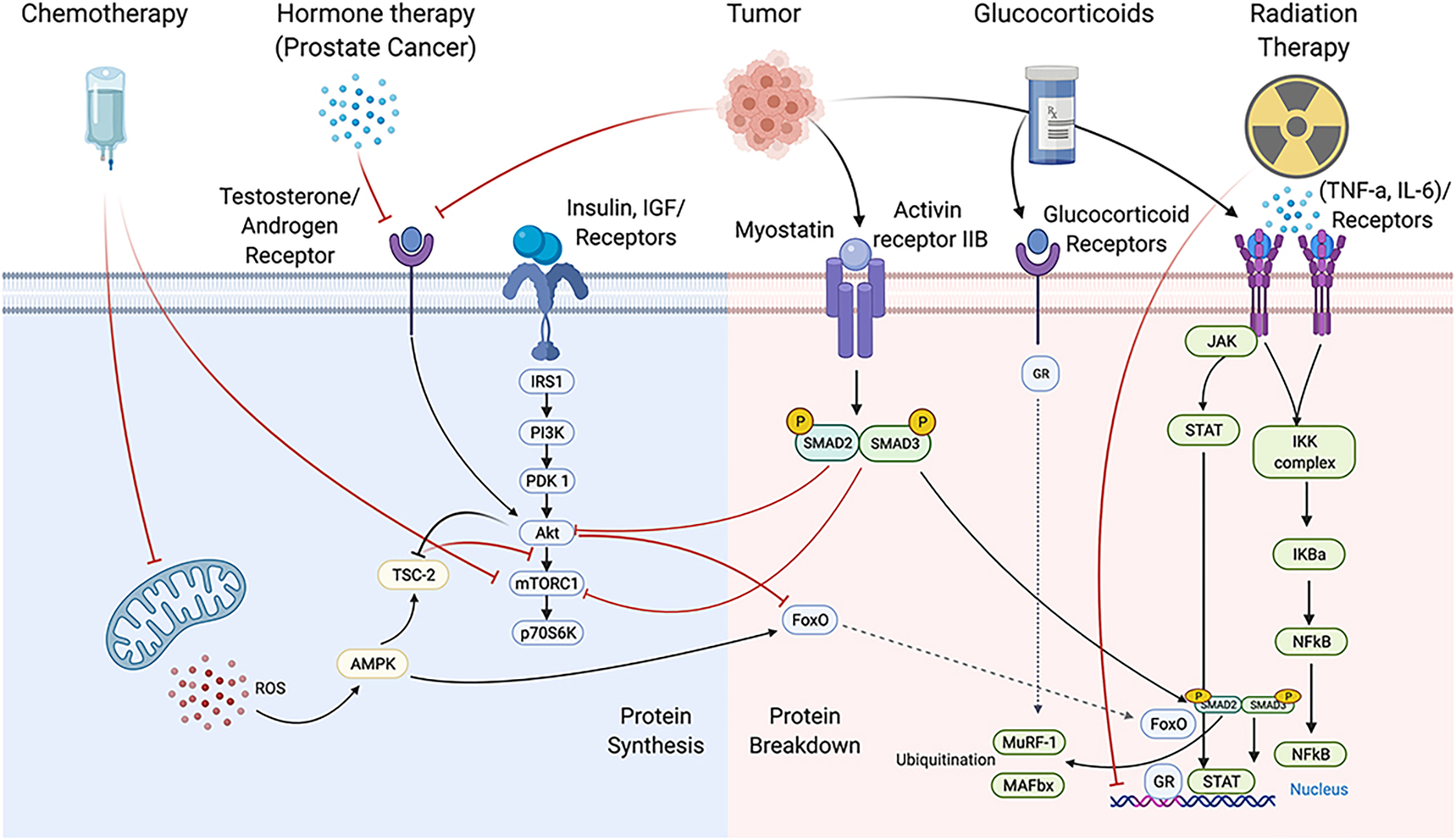
Overview of the impact of various cancer treatments on anabolic and catabolic signalling. Skeletal muscle mass maintenance relies primarily on the balance of protein synthesis and protein degradation, of which there is an overlap of signalling pathways. Protein synthesis relies heavily on activation of mTORC1 activation of p70S6K and suppression of E3ligase activation through FoxO. The type/dose of therapy (chemotherapy, radiation, hormone, etc.) have robust and distinct impacts on intracellular regulators of skeletal muscle mass such as mTOR, androgen receptor, myostatin, and NF-kB signalling; however, these mechanisms continue to be unearthed. Created with biorender.com.

As outlined above, different types/doses of anticancer therapy can have differential effects and mechanisms influencing muscle loss in cancer. This is exacerbated in clinical settings, where individuals with cancer often receive a variety of therapies that compound the treatment-related burden and potentially, loss of muscle mass. Further, more aggressive or advanced tumours may require more intensive treatment regimens for treatment and control. It has been recently demonstrated that the degree of skeletal muscle loss and potential reversibility is influenced by the intensity/burden of systemic treatment for metastatic colorectal cancer.^107^ Further, differential loss of skeletal muscle mass has also been demonstrated in individuals with cancer receiving chemotherapy vs. those receiving TKIs, (loss of muscle mass was lowered with TKIs than chemotherapy).^108^ Taken collectively, these data support that some of the factors affecting muscle loss in cancer may be attributed to the type and overall burden of various anticancer therapies. Importantly, the loss of skeletal muscle mass during treatment may also be associated with increased treatment-related toxicities, which could further exacerbate muscle loss, and present additional barriers to exercise participation (Figure 1).^6,108,109^

## More than a signal: Additional barriers to exercise participation and adaptations

### Malnutrition

Beyond the above-mentioned impact on signalling pathways, the receipt of different anticancer therapies can exacerbate catabolic processes through various side effects and large caloric deficits.^110,111^ Vomiting, nausea, anorexia, xerostomia, dysphagia, oral ulcerations/mucositis, and pain are all common concerns during various treatments. In turn, this leads to reduced food intake, expansion of an energy deficit, and resultant weight loss, that is, exacerbated cachexia.^11,111,112^ Further, cancers of the head and neck receiving surgery and chemoradiation often require enteral or parenteral nutrition to overcome challenges to eating and consuming food.^113^ Malabsorption is also a common concern in certain tumour types (e.g. gastro-oesophageal and pancreatic) where tumour location and different treatments may result in a reduced ability to absorb nutrients.^112^ Malnutrition in cancer is both complex and multifactorial, and likely a consequence of a multitude of factors including tumour type, treatment(s), psychology, and inter-individual considerations.^110^ Nevertheless, the accompanying deficits in protein and total energy intake contribute to the loss of bodyweight and muscle loss across the cancer continuum. Importantly, these also present barriers to (i) consuming adequate energy to tolerate exercise sessions and (ii) receiving appropriate nutrient intake and composition to overcome excess catabolism or promote recovery/adaptation from exercise training.

### Comorbidities, injuries, exercise tolerance, and compliance

**Figure 2:**
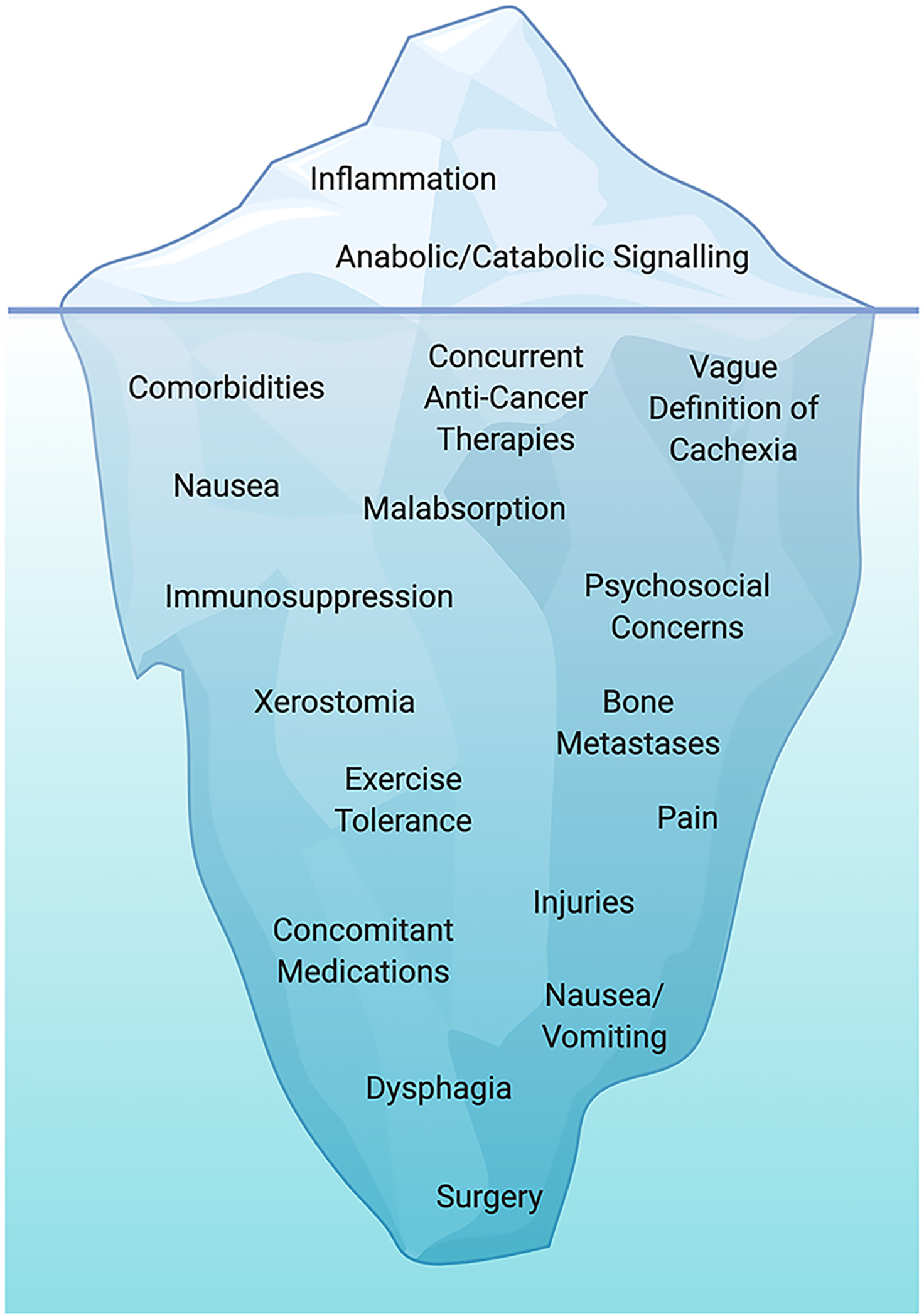
Challenges to overcoming cachexia in clinical settings. The majority of pre-clinical literature to date has focused on targeting various inflammatory cytokines and anabolic/catabolic signalling pathways. There have been some positive results, although translation into clinical settings brings about a series of additional barriers and challenges to overcome. A variety of tumour-related/treatment-related factors (location of tumour, type of treatment, etc.) and side effects (pain, nausea, vomiting, dysphagia, and oral ulcerations) can result in malabsorption of nutrients and/or malnutrition. This can bring challenges to consume adequate/appropriate nutrients to (i) maintain body weight and muscle mass, (ii) fuel adequately to tolerate exercise sessions, and (iii) recover adequately from and adapt to habitual exercise training. Fatigue, pain, comorbidities, immunosuppression, injuries, and presence of bone metastases can affect both the exercise prescription and the ability to tolerate a sufficient load/volume of exercise training to promote positive muscular adaptation. The presence of concurrent treatments, recovery from surgical procedures, and/or concomitant medications may affect the ability to received and respond to an appropriate training dose. Created with biorender.com.

The hypothesis that exercise is sufficient to overcome many of the catabolic processes involved in the loss of muscle mass relies on a weighty assumption that the individual will be able to tolerate a training programme that is sufficient to ameliorate muscle loss. There are, however, numerous documented conditions impacting a patient’s ability to participate in, adhere to, and respond to an appropriate training stimulus. Other tumour-related or treatment-related side effects such as peripheral neuropathy, dyspnoea, nausea, pain, anaemia, neutropenia, and bone metastases can impact the ability to perform exercise safely, particularly at the load, dose, or frequency desired to target muscle mass (Figure 2).^114^

In solid tumours, surgical procedures to remove the cancer may result in prolonged recovery and/or impairment to surrounding muscles/joints that limit the ability to perform exercise appropriately. Further, in ageing populations, comorbidities such as obesity, hypertension, arthritis, and arterial diseases are not uncommon.^115^ The presence of comorbidities (and their medications) can compound challenges to exercise tolerance and ensuring individuals can receive the appropriate training stimulus for reversal of muscle loss. For example, the results of a recent randomized controlled trial (RCT) demonstrated that metformin (a common medication for type II diabetes) blunted the hypertrophic response to resistance training in older adults.^116^ Consequently, beyond the presence of side effects and comorbidities impacting the tolerance to exercise, medications taken for these comorbidities may also inhibit exercise adaptations.

Disease progression that results in metastases to the bone also represents unique challenges to exercise prescription and loading.^114,117,118^ Osteolytic lesions can accelerate bone resorption at the specific site of metastases and increase the risk of fractures, whereas osteoblastic lesions can result in the deposition of new bone at lesion sites, often resulting in burdensome pain. Individuals with cancer can present with predominantly osteolytic (i.e. breast cancer and multiple myeloma) or osteoblastic lesions (i.e. prostate cancer and Hodgkin lymphoma) or a combination of both (i.e. breast, gastrointestinal, and squamous cancers), which present challenges in the configuration of exercise programmes.^119,120^ There is mounting evidence demonstrating the safety and feasibility of exercise that is specifically tailored to this patient population.^114,117,118^ However, these exercise programmes thus far have been extensively modified, with either the avoidance of loading, or by using isometric bodyweight loaded exercises at the lesion sites. Consequently, whether an exercise programme that would be sufficient to prevent muscle loss in advanced cancers with muscle loss is currently unclear.

Importantly, it is unknown if individuals experiencing muscle loss in cancer will have the same response to exercise as what would be expected from apparently healthy counterparts. Namely, exercise programmes to promote hypertrophy of skeletal muscle require a high degree of effort, performing exercises at a sufficiently high load and/or at a close proximity to failure.^121^ Exercise interventions in clinical settings are notoriously complex, compounded by the simultaneous delivery of multimodal cancer treatment, presence of comorbidities and/or limited performance status, not to mention the logistical challenges of delivering exercise in clinical settings.^114^ Therefore, some individuals may not be able to achieve the appropriate type, dose, or frequency of exercise required to sufficiently target muscle mass, particularly in a short enough timeframe to confer a clinically meaningful benefit. An overview of some of the considerations and challenges in conducting exercise trials to target muscle wasting is provided in Table 1.

Reviewing the tumour-induced and cancer treatment-induced muscle loss from a molecular biology perspective, there is a clear rationale whereby exercise can target key signalling pathways to prevent the incidence or progression of muscle loss. There are ongoing trials that are likely to further expand our understanding of the role of exercise in the management of muscle loss in cancer (ClinicalTrials.gov Identifier: NCT04131426 & NCT02330926; ANZCTR.org.au Identifier: ACTRN12619000426189). These trials utilize a combination of personalized exercise with additional support (nutrition and/or anti-inflammatories),^133–135^ highlighting a recognition from the field that multimodal therapy is likely to be a more appropriate approach to target muscle loss. As the results of these trials emerge, our understanding of the role of exercise in the management of muscle loss in cancer will evolve. It is exciting to hypothesize the potential for exercise to overcome muscle loss in cancer. However, expectations should also be tempered, particularly in absence of evidence, to the very likely possibility that there will be limitations to the extent of its efficacy/effectiveness of exercise in these settings.

### Sex differences in muscle wasting

An interesting and exciting area of research is emerging, indicating sex differences in the regulation of muscle wasting in cancer. Importantly, a thorough discussion of the biological sex differences in muscle wasting in cancer is beyond the scope of this paper, and they have been expertly reviewed previously.^136,137^ Briefly, it has been posited that differences in muscle fibre content, protein turnover, satellite cell content, hormone concentration and interactions, and mitochondrial content may all contribute to differential mechanisms of muscle loss between male and female participants.^138^ There is a need for clinical research investigating the rates, magnitude, and mechanisms of muscle loss in cancer to determine if differences exist according to biological sex, which might shed light on the need for unique interventions.

## Perspective on critical areas of future research

### Understanding the mechanisms of treatment-induced muscle loss and response to exercise in humans

As expertly outlined by Dolly *et al*., the translation of pre-clinical evidence to humans is notoriously difficult and often not replicated.^12^ In pre-clinical models, the homogeneity of subjects and ability to control for confounding factors (i.e. diet, exercise, and additional therapies/medications) is a clear advantage to understanding specific mechanisms for muscle loss in cancer.^63,139^ The human model of cancer-related muscle loss is inarguably more complex, with a variety of phenotypical, treatment, psychological (state and trait), lifestyle, energy balance, and dietary factors. Additionally, individuals often present with comorbidities (i.e., diabetes, cardiovascular disease, and COPD) and medications that compound outcome variability. Albeit a strength of pre-clinical research, one of the most difficult elements of research in humans with cancer is the recruitment of a homogenous group. Dolly *et al*. demonstrated that despite increased proteolysis and mitochondrial dysfunction being reported in pre-clinical models, these were not apparent in numerous clinical studies of cancer cachexia.^12^ Further, the same concept applies when aiming to study exercise adaptations in this context, whereby replicating the dynamic and complex variables involved in resistance exercise prescription in animal models is almost impossible. Consequently, there is a need to investigate how tumour-related and treatment-related factors impact the muscle signalling and morphology that is housed in complex, human biological systems.

### Need for reference groups of age-/sex-matched healthy controls without history of treatment

To fully understand the impact of cancer and its treatments on skeletal muscle biology, and its ability to respond and adapt to training stimuli, there is a need for comparison with non-cancer controls. Judge *et al*. (2018) profiled the skeletal muscle phenotype in individuals with pancreatic cancer vs. a weight-stable non-cancer control.^140^ Individuals with a bodyweight loss greater than 5% in the previous 6 months were considered cachectic (*n* = 15). Compared with non-cachectic individuals and non-cancer controls, cachectic individuals exhibited greater skeletal muscle fibrosis, in addition to evidence of pathology by abnormal fibre membranes, increased numbers of mononuclear cells, and increased connective tissue surrounding myofibers.^140^ Interestingly, the biopsies were obtained from individuals at the beginning of surgery, and no differences in collagen content were seen in individuals with and without neoadjuvant therapy, indicating that neither surgery nor therapy can explain the pathologies and that other factors might be at play. Despite the limited sample size, this study highlights the value in non-cancer controls in understanding the pathological impact of cancer and its treatments on skeletal muscle. Herein, these comparisons will be able to offer more of an insight into (i) the impact of cancer treatments on muscle physiology and (ii) how these changes (if any) impact the potential for skeletal muscle to repair and regenerate following exercise training.

### How long aberrations in muscle signalling and pathology persist after treatment

Low muscle mass is associated with frailty, functional decline, recurrence, and cancer-related mortality.^2,141^ As such, there is a rapidly growing area of research, investigating various exercise, nutrition, and pharmacological interventions to arrest and reverse the loss. If signalling pathways and morphological properties are impaired with treatment, these may impact the ability to receive and respond to these interventions. Importantly, it is not well understood how long these symptoms persist after treatment. Long-term follow ups of individuals who have received treatment for child/young adult cancer reveal persistent impairments in muscular function and tissue decline.^105^ However, much less is known of the persistent and/or late effects of cancer and its therapies on signalling pathways once treatment has finished. In adult cancers, untangling the impact of natural ageing, diet, and inactivity on these pathways, vs. the sustained, or late effects of treatments is difficult.

### An adjunct to pharmacotherapy?

There is continued interest in the investigation of pharmacological compounds to target muscle loss. Certainly, there is evidence to support that some of these agents can support muscle growth. In a pre-clinical model, a myostatin receptor (ActRIIB) prevented muscle loss and prolonged survival without altering fat mass or tumour growth across different cachexia models.^142^ In clinical research, anamorelin (a ghrelin receptor agonist) has been demonstrated to increase muscle mass in non-small cell lung cancer.^143,144^ However, most clinical trials with pharmacology have demonstrated increases in mass, with mixed/scarce evidence for improvements in function.^145,146^ Ironically, the majority of the extant literature in clinical exercise oncology indicates that exercise can result in meaningful changes in physical function but divergent results for mass.^8,147^ Herein represents a critical opportunity for investigations to combine exercise with pharmacology as a multimodal approach in muscle loss research. Specifically, the addition of an appropriately structured exercise programme to pharmacological interventions may result in potential synergistic or additive effects on both muscle mass and physical function.

## Conclusions

Muscle loss in cancer is not a homogenous process, and while there may be commonalities, it is likely that the signalling pathways and morphological properties affected will be different within and across cancer types and treatment modalities. Moreover, even less is known about the potential synergistic effects of cancer, its treatments, and the different pathways impacting muscle loss. As such, targeting a single pathway in a clinical setting with complex human biology remains an uphill battle. An infinite number of questions remain regarding the clinical applications of exercise to target muscle loss, not least the feasibility of intervening across different tumour types and those with more aggressive/advanced diseases. Given the hurdles faced for skeletal muscle growth in individuals with cancer, preservation of muscle, particularly if improvements in other comorbidities and physical function are seen, is likely to be a successful outcome.

## Figures and Tables

**Table 1:** Considerations and challenges in clinical research of exercise in muscle wasting

Considerations	Overview

Methodological considerations	
Operational definition of cachexia	Sarcopenia historically referred to low muscle mass, although most recent consensus from working groups have both recognized and promoted low muscle strength/function as a key component of this condition.^1,122^ Cachexia is generally categorized into three distinct phases: pre-cachexia, cachexia, and refractory cachexia (where life expectancy is <3 months). More recently, the operational definition has included >5% unintentional weight loss in the past 6 months, or 2–5% weight loss with either a body mass index of <20 kg/m^2^,^3^ or reduced muscle mass. Perhaps most important is the difficultly in clinically diagnosing muscle loss conditions in addition to being able to distinguish them between each other. These definitions are somewhat vague and make it difficult to accurately diagnose and intervene appropriately on the condition. Ultimately, the aetiology and accurate diagnosis (in addition to treatment burden) will provide insight into the complexity, severity and potentially reversibility of the condition through exercise (and/or nutrition and pharmacology) interventions.
Measurement & outcome considerations	There are ongoing conversations regarding what constitutes ‘success’ in muscle loss in cancer research. The United States Food and Drug Administration has traditionally required a dual endpoint of changes in muscle mass and function, although recently has accepted a composite endpoint including quality of life and body weight gain. Other outcomes of interest to researchers include symptom management, body-weight maintenance or reversal, appetite, nutritional status, physical function, and muscle strength, in addition to mortality.^1,10,11,123–126^ It is also likely that successful outcomes will depend on the operational definition of muscle loss, along with the time and type of intervention.
Rigour & transparency	There is a clear need for research designed with greater rigour and transparency. Appropriately powered trials with low risk of bias,^127^ clearly defined primary and secondary outcomes that preregistered trials with sufficient detail (i.e. outcome score and analysis plan for outcomes),^128^ transparent of protocol deviations, and reporting of statistical plan and open sharing of data can all serve to enhance the quality of research in this area.
Time of intervention	Ideally, the best time to intervene on muscle wasting would be before it has begun to occur. However, numerous challenges to this exist, including its often-insidious nature (making it difficult to detect changes early), in addition to logistical challenges of attempting an intervention at a point of diagnosis, where individuals may be consumed by the emotional, financial and time burden of a diagnosis and treatment schedule.
Condition or tumour type?	A challenge to clinical research in the area of cachexia is whether to refine an intervention to fit a specific tumour type with cachexia, to limit potential confounding variables, or to expand to include individuals with/at risk of muscle wasting, regardless of tumour-related and treatment-related factors.
Investigating sex differences	There is emerging evidence that sexual dimorphism exists in muscle wasting, with male and female participants exhibiting unique differences that affect the microenvironment and signalling in skeletal muscle. As research in this area continues to grow, sex differences in muscle wasting may require distinct and unique interventions.
Comparison with non-cancer controls	There is increasing interest in distinguishing cancer-related muscle loss from apparently healthy ageing.^129,130^ Comparing rates of muscle loss, in addition to therapeutic interventions in cancer relative to non-cancer controls, can help better understand and isolate the magnitude, severity, and degree of reversibility of muscle loss as a consequence of cancer and its treatments.
Concurrent anticancer therapies	Most of the pre-clinical work investigates the impact of cancer treatments on muscle wasting using single modality therapies. Translating these findings into clinical models where individuals often receive different combinations of treatments can be challenging. As clinical research continues to evolve, as will our understanding of concurrent treatments on muscle loss and the degree of reversibility through exercise.
Immunosuppression	Suppression of immune function may impact the ability to participate in and respond to an exercise programme. Careful consideration should be paid to the type and dose of exercise during
Concomitant medications	Medications being taken in addition to those for cancer (i.e. metformin for diabetes) may blunt the adaptations from exercise.
Nutritional challenges	
Nausea/Vomiting	Nausea and vomiting can impact the ability to participate in an exercise programme through illness. Additionally, vomiting may indirectly result in a reduced energy consumption and an ability to receive the appropriate nutrients to fuel and respond to an exercise session.
Dysphagia/Xerostomia	Dysphagia (difficulty swallowing) and xerostomia (dry mouth) can affect the desire and ability to consume sufficient nutrients through habitual diet.
Malnutrition	A variety of factors can result in a reduced/insufficient nutritional intake in cancer. While parenteral and enteral nutritional strategies may be indicates, individuals may have difficulty consuming sufficient calories to support an exercise programme targeting muscle mass.
Malabsorption	Certain cancers such as gastroesophageal, liver, and pancreatic cancer are at a heightened risk of malabsorption, potentially impacting their ability to utilize nutrients appropriately to fuel and respond to an exercise session.
Exercise considerations	
Bone metastases	The type and extent of bone metastases can dramatically impact the exercise prescription. While current evidence indicates preliminary safety of exercise in individuals with bone metastases, whether they can exercise at a level that is sufficient to promote the maintenance or increase in muscle with cachexia is unclear.
Dyspnoea	Dyspnoea (shortness of breath) may limit exercise capacity and the ability to perform exercise at a sufficient dose to facilitate adaptations.
Pain	Cancer-related pain is not uncommon and can impact the specific exercise selection and potentially the volume/intensity of exercise that would be tolerable. Additionally, high levels of pain may impact motivate to participate in an exercise programme.
Cancer-related fatigue	Current evidence indicates that individuals who are most fatigued stand to benefit the most from an exercise programme. However, extensive fatigue may impact the motivation to participate in an exercise programme, and the overall training volume that could be achieved.
Comorbidities	Individuals with diabetes, COPD, or cardiovascular disease may require additional exercise modifications that preclude them from attaining a sufficient dose to offset muscle wasting.
Depression & anxiety	Individuals experiencing depressive symptoms or anxiety related to their cancer may experience mood disruptions and reduced motivation to participate in and exercise programme of sufficient dose and duration to illicit positive adaptations.^131,132^
